# Tenofovir treatment augments anti-viral immunity against drug-resistant SIV challenge in chronically infected rhesus macaques

**DOI:** 10.1186/1742-4690-3-97

**Published:** 2006-12-21

**Authors:** Karin J Metzner, James M Binley, Agegnehu Gettie, Preston Marx, Douglas F Nixon, Ruth I Connor

**Affiliations:** 1 Aaron Diamond AIDS Research Center and The Rockefeller University, New York, NY 10016, USA; 2 Torrey Pines Institute for Molecular Studies, San Diego, CA 92121, USA; 3 Tulane Regional Primate Research Center and Department of Tropical Medicine, Tulane University Health Sciences Center, Covington, LA 70433, USA; 4 University of California, San Francisco, Department of Medicine, Division of Experimental Medicine, San Francisco, CA 94110, USA; 5 University of Erlangen-Nuremberg, Institute of Clinical and Molecular Virology, Schlossgarten 4, Erlangen, 91054, Germany; 6 Department of Microbiology and Immunology, HB7556, Dartmouth-Hitchcock Medical Center, One Medical Center Drive, NH 03756, Lebanon

## Abstract

**Background:**

Emergence of drug-resistant strains of human immunodeficiency virus type 1 (HIV-1) is a major obstacle to successful antiretroviral therapy (ART) in HIV-infected patients. Whether antiviral immunity can augment ART by suppressing replication of drug-resistant HIV-1 in humans is not well understood, but can be explored in non-human primates infected with simian immunodeficiency virus (SIV). Rhesus macaques infected with live, attenuated SIV develop robust SIV-specific immune responses but remain viremic, often at low levels, for periods of months to years, thus providing a model in which to evaluate the contribution of antiviral immunity to drug efficacy. To investigate the extent to which SIV-specific immune responses augment suppression of drug-resistant SIV, rhesus macaques infected with live, attenuated SIVmac239Δnef were treated with the reverse transcriptase (RT) inhibitor tenofovir, and then challenged with pathogenic SIVmac055, which has a five-fold reduced sensitivity to tenofovir.

**Results:**

Replication of SIVmac055 was detected in untreated macaques infected with SIVmac239Δnef, and in tenofovir-treated, naïve control macaques. The majority of macaques infected with SIVmac055 experienced high levels of plasma viremia, rapid CD4^+ ^T cell loss and clinical disease progression. By comparison, macaques infected with SIVmac239Δnef and treated with tenofovir showed no evidence of replicating SIVmac055 in plasma using allele-specific real-time PCR assays with a limit of sensitivity of 50 SIV RNA copies/ml plasma. These animals remained clinically healthy with stable CD4^+ ^T cell counts during three years of follow-up. Both the tenofovir-treated and untreated macaques infected with SIVmac239Δnef had antibody responses to SIV gp130 and p27 antigens and SIV-specific CD8^+ ^T cell responses prior to SIVmac055 challenge, but only those animals receiving concurrent treatment with tenofovir resisted infection with SIVmac055.

**Conclusion:**

These results support the concept that anti-viral immunity acts synergistically with ART to augment drug efficacy by suppressing replication of viral variants with reduced drug sensitivity. Treatment strategies that seek to combine immunotherapeutic intervention as an adjunct to antiretroviral drugs may therefore confer added benefit by controlling replication of HIV-1, and reducing the likelihood of treatment failure due to the emergence of drug-resistant virus, thereby preserving treatment options.

## Background

Initiation of antiretroviral therapy (ART) in patients with HIV-1 infection can rapidly reduce plasma viremia, bolster immune responses, and improve clinical outcome [1-3]. Despite significant progress in the clinical management of HIV-1 infection, the therapeutic efficacy of ART is often undermined by incomplete suppression of virus replication and the emergence of drug-resistant HIV-1 [4]. Drug-resistant strains of HIV-1 harbor mutations that can negatively impact viral fitness, but these viruses gain a replicative advantage in the presence of drug and can be associated with treatment failure and clinical progression [5,6]. Moreover, drug-resistant HIV-1 can be transmitted to treatment-naïve individuals, thereby limiting the range of therapeutic options available to these patients [7,8].

The extent to which HIV-specific immune responses suppress the emergence of drug-resistant strains is not well understood, but may be influenced by immune recognition of epitopes containing key resistance mutations. CD8^+ ^T cells from individuals harboring multi-drug-resistant HIV-1 still respond *in vitro *to proteins and peptides containing commonly found drug resistance mutations [9,10], suggesting that immune recognition is adaptive and responsive to the emergence of drug-resistant virus. Whether these responses control replication of drug-resistant HIV-1 *in vivo*, and whether they can be induced in HIV-infected patients as a protective measure against the emergence of drug-resistant viral variants is unknown.

The concept that drug efficacy can be augmented by strong antiviral immune responses is compelling, and has led to efforts to stimulate antiviral immunity in HIV-infected patients on ART. Various immunotherapeutic strategies including structured treatment interruptions, therapeutic immunization, and immunomodulatory agents have been explored with limited success to date [11], and serve to highlight the complexity of the interaction between host immunity, virus replication and drug efficacy.

In this respect, animal studies using SIV infection of non-human primates provide a useful tool to shed light on the mechanisms of immune-mediated control of infection, the impact of antiretroviral drugs on virus replication [12], and the emergence and evolution of drug-resistant variants [13]. SIV infection in rhesus macaques shares many of the immunopathogenic features of HIV-1 infection in humans, and this model has been used to evaluate the contribution of antiviral immune responses to suppression of virus replication during ART intervention. *In vivo *depletion of CD8^+ ^T cells in SIV-infected macaques receiving treatment with the reverse transcriptase (RT) inhibitor tenofovir {9-[2-(phosphonomethoxy)propyl] adenine, PMPA} leads to an increase in viremia, providing direct evidence that these cells significantly contribute to the success of tenofovir in suppressing replication of virulent SIV [14].

The notion that antiviral immune responses play a critical role in augmenting the efficacy of ART is amenable to further study in rhesus macaques infected with live, attenuated SIV, in which broad SIV-specific cellular and humoral immune responses are induced, and can confer robust protection against exogenous SIV challenge [15]. Interestingly, antiviral immunity in these animals fails to fully control replication of the endogenous attenuated SIV strain and infected macaques remain continuously viremic for periods of months to years [16]. *In vivo *depletion of CD8^+ ^T cells in macaques infected with live, attenuated SIV leads to a marked increase in viremia indicating a critical role for these cells in controlling virus replication [17]. In the absence of drug intervention, pathogenic sequelae can develop in both neonatal [18] and adult [19] animals infected with live, attenuated SIV, mirroring in certain aspects the clinical progression of chronic HIV-1 infection in humans, including increasing viral burden and progressive loss of CD4^+ ^T cells.

The immunologic and virologic features of macaques infected with live, attenuated SIV, typified by low-level viremia and strong SIV-specific humoral and cellular immune responses, provide a unique opportunity to examine the contribution of SIV-specific immunity to augmenting ART and suppressing replication of drug-resistant virus during chronic infection. Here, we report on treatment of macaques chronically infected with SIVmac239Δnef with a short-term regimen of tenofovir and challenged with drug-resistant SIV. Our results indicate that tenofovir given to macaques with established anti-viral immunity can prevent replication of drug-resistant virus in the setting of chronic SIV infection.

## Results

### Effect of tenofovir on SIVmac055 challenge of naïve rhesus macaques

**Figure 1:**
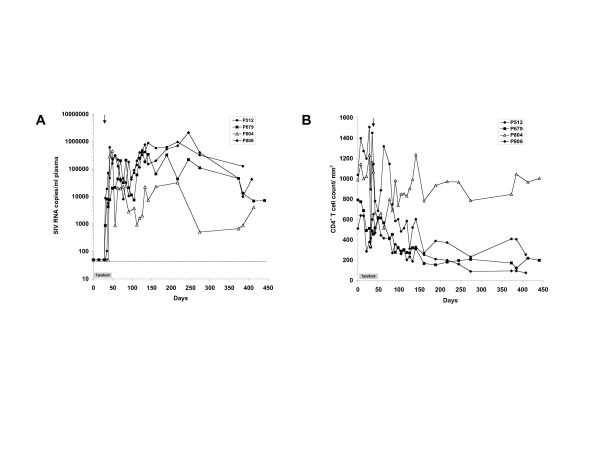
**Pre-treatment of naïve rhesus macaques with tenofovir and subsequent infection with SIVmac055**. Adult rhesus macaques were treated for 4 weeks with tenofovir at a dose of 30 mg/kg body weight, and then inoculated intravenously with SIVmac055 on day 28 (arrow). Tenofovir treatment was continued for an additional 2 weeks after SIVmac055 infection. Virus replication and CD4^+ ^T cell counts were monitored for > 1 year of follow-up. (A) Plasma viral load was measured by real-time PCR with a sensitivity of 50 SIV RNA copies/ml, (B) CD4^+ ^T-cell counts.

To evaluate the dose and replicative capacity of SIVmac055 in the presence of tenofovir, four drug-naïve adult rhesus macaques were subcutaneously given tenofovir daily for 4 weeks prior to intravenous inoculation with 10^4 ^TCID_50 _of SIVmac055. Tenofovir treatment was continued for an additional 2 weeks after SIVmac055 inoculation, and virus replication, CD4/CD8 T cell counts, and clinical adverse events were monitored at regular intervals (Fig. 1). All of the macaques had CD4^+ ^and CD8^+ ^T cell counts within the normal range at baseline (Table 1). Tenofovir treatment was well tolerated in the macaques and no sustained changes in CD4^+ ^and CD8^+ ^T cell counts were observed during the treatment intervention, with the exception of one macaque (P679) that experienced a transient drop in CD4^+ ^T cell counts. None of the macaques exhibited any serious adverse events associated with tenofovir treatment.

**Table 1:** Immunization history and baseline characteristics of rhesus macaques prior to tenofovir treatment

			**SIVmac239Δnef proviral load**		**Lymphocyte counts (cells/mm^3^)^d^**		**SIVmac239Δnef plasma viral load^e^**
**Macaque**	**SIV infection^a^**	**Protection against SIVmac251 challenge^b^**	**DNA copies/10^6 ^genomic equivalents^c^**	**Tenofovir**	**CD4^+ ^T cells**	**CD8^+ ^T cells**	**(RNA copies/ml plasma**
P512	-	ND	ND	+	1039	705	< 50
P679	-	ND	ND	+	793	609	< 50
P804	-	ND	ND	+	989	847	< 50
P806	-	ND	ND	+	512	371	< 50
1494	SIVmac239Δnef	+	29 ± 23	-	558 ± 21	520 ± 75	1.5 × 10^3^
1512	SIVmac239Δnef	+	14 ± 9	-	868 ± 207	864 ± 269	5.5 × 10^2^
1488	SIVmac239Δnef	+	66 ± 41	+	725 ± 82	753 ± 208	< 50
1498	SIVmac239Δnef	+	12 ± 10	+	669 ± 61	1471 ± 343	3.5 × 10^3^
1514	SIVmac239Δnef	+	291 ± 123	+	703 ± 180	963 ± 342	< 50

^a ^Macaques were infected with SIVmac239Δnef approximately 3 years prior to initiation of this study [16]

^b ^Challenge with SIVmac251 was carried out from 10 and 25 weeks after immunization with SIVmac239Δnef [16]

^c ^Mean ± standard deviation of 5–7 time points within 2.5 years before administration of tenofovir

^d ^Mean ± standard deviation of days -14, -6 and 0 before administration of tenofovir

^e ^Day 0 before administration of tenofovir

**Figure 2:**
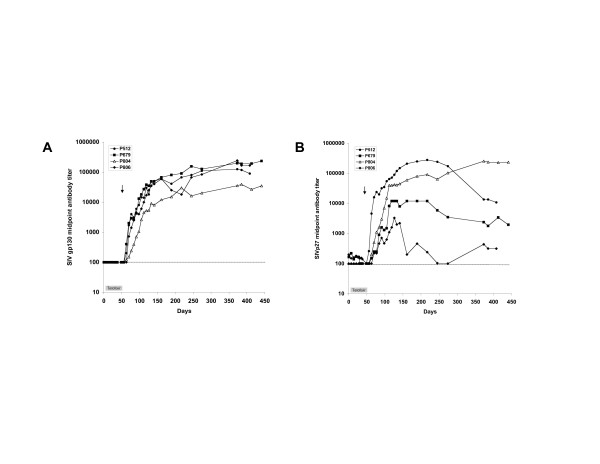
**SIV-specific antibody responses in tenofovir-treated macaques infected with SIVmac055**. Plasma antibody titers to (A) SIV gp130 and (B) SIV p27 were measured during tenofovir treatment (days 0–42) and after challenge with SIVmac055 (day 28, arrow). Data is expressed as the midpoint antibody titer based on serial titration of plasma and antibody detection by antigen-specific ELISA [16, 41].

Quantification of SIV RNA by real-time PCR revealed the presence of SIVmac055 RNA in the plasma of all 4 macaques within 3 to 14 days of inoculation (Fig. 1A). Peak viremia occurred between days 14 and 42 post-infection with maximal plasma viral loads ranging from 2 – 6.3 × 10^5 ^SIV RNA copies/ml plasma. Viral loads remained elevated, and 3 of 4 macaques developed symptoms of simian AIDS and were euthanized within 12 to 15 months after infection. These macaques experienced a significant decline in CD4^+ ^T cells associated with SIVmac055 infection (Fig. 1B) and displayed typical clinical and pathological features consistent with simian AIDS. The remaining macaque (P804) became infected with SIVmac055, but was able to control the infection and CD4^+ ^T cells remained stable in this animal for over a year (Fig. 1B). However, macaque P804 was subsequently euthanized due to severe self-inflicted trauma. Upon autopsy, no signs of simian AIDS were found, although tissues were not examined for the presence of SIV. All four animals developed SIV-specific anti-gp130 and anti-p27 antibodies, but no significant differences in antibody titers were seen between the three macaques that developed simian AIDS and macaque P804 who appeared to control SIVmac055 infection (Fig. 2A and 2B). Taken together, these results demonstrate that SIVmac055, when inoculated intravenously at a dose of 10^4 ^TCID_50_/ml, is able to infect and replicate in the majority of rhesus macaques receiving concurrent antiretroviral treatment with tenofovir.

### Analyses of SIVmac055 nucleotide sequences in control rhesus macaques

Five-fold resistance to tenofovir *in vitro *is associated with a K65R mutation, and additional compensatory mutations, in the RT gene of SIVmac055 [20]. To determine whether these mutations were present in virus isolated from the tenofovir-treated macaques, plasma viral RNA was reverse transcribed, amplified with oligonucleotides spanning part of the SIV *pol *gene, and the PCR products directly sequenced. Five mutations in RT are reported for SIVmac055: K65R, N69T, R82K, A158S, and S211N [20]. Sequence analyses of the SIVmac055 stock used in our experiments revealed these five mutations and an additional mutation (K64R) present in a minor population of variants (data not shown). All five mutations associated with tenofovir resistance were identified in virus sequences from the tenofovir-treated macaques infected with SIVmac055, and these mutations were stable over time (Table 2). In all animals, a mixed population (S211N/S211S) was found at week 35 post-infection and persisted thereafter. The K64R mutation was present in three animals at week 2 post-infection as mixed population (K64R/K). By week 35, the K64R mutation emerged as the major population in three infected animals.

**Table 2:** Mutations in plasma SIV RT from rhesus macaques infected with SIVmac055

		**RT Mutations**						
		
**Macaque**	**Week**	**I31**	**K64**	**K65**	**N69**	**R82**	**A158**	**S211**
P512	2	-	R/K	R	T	K	S	N/S
	6	-	R/K	R	T	K	S	N
	35	-	R/K	R	T	K	S	N/S
	55	-	R	R	T	K	S	N/S
P679	2	-	R/K	R	T	K	S	-
	23	-	R/K	R	T	K	S	-
	35	-	R	R	T	K	S	N
	55	-	R	R	T	K	S	N/S
P804	2	-	R/K	R	T	K	S	N
	11	-	R	R	T	K	S	N
	35	-	-	-	T	K	S	N/S
P806	2	-	-	R	T	K	S	N/S
	11	V/I	-	R	T	K	S	N/S
	35	V	R/K	R	T	K	S	N/S
	55	V	R/K	R	T	K	S	N/S

### Administration of tenofovir to macaques with chronic SIVmac239Δnef infection

Five adult rhesus macaques were infected with SIVmac239Δnef approximately three years prior to initiation of this study [16]. All five were shown to resist pathogenic SIVmac251 challenge with no evidence of SIVmac251 RNA or DNA in either plasma or lymph nodes over a 3-year follow-up period [16]. However, all the animals remained intermittently viremic with low levels of plasma SIVmac239Δnef detected throughout the follow-up period, consistent with a failure to fully suppress replication of the original infecting strain. Each of the animals developed robust SIV-specific humoral and cellular immune responses, which may have contributed to protection from exogenous SIVmac251 challenge, but these responses were insufficient to prevent ongoing replication of the endogenous attenuated virus. All of the SIVmac239Δnef-infected macaques had CD4^+ ^and CD8^+ ^T cell counts within the normal range, and plasma viral loads ranging from < 50 - 3.5 × 10^3 ^SIV RNA copies/ml plasma immediately prior to initiation of this study (Table 1).

**Figure 3:**
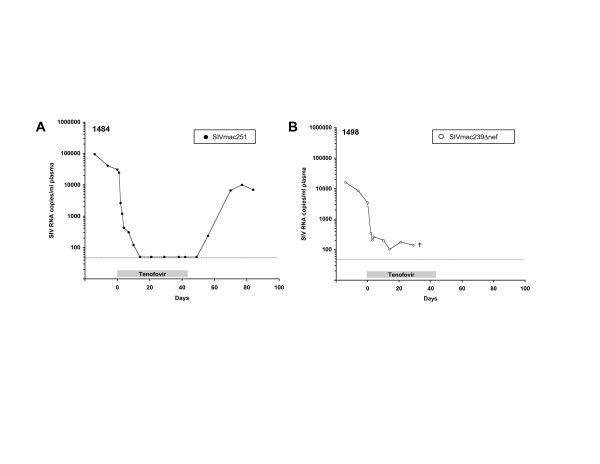
**Effect of tenofovir on plasma viral load in macaques infected with SIVmac251 or SIVmac239Δnef**. Rhesus macaques with chronic SIV infection were treated for 6 weeks with tenofovir at a dose of 30 mg/kg body weight. The effect on SIV replication was determined by quantification of plasma SIV RNA by allele-specific real-time PCR. Plasma viral load is expressed as SIV RNA copies/ml and shown for macaques with replicating (A) SIVmac251 and (B) SIVmac239Δnef.

**Figure 4:**
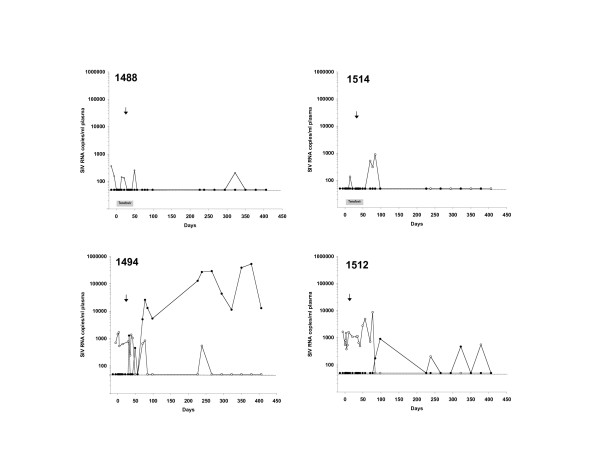
**Replication of SIVmac055 in tenofovir-treated and untreated macaques infected with SIVmac239Δnef**. Macaques chronically infected with SIVmac239Δnef were treated for 4 weeks with tenofovir (1488, 1514) or left untreated (1494, 1512). Both treated and untreated macaques were challenged with SIVmac055 on day 28 (arrow). Replication of SIV was measured by allele-specific PCR to discriminate between SIVmac055 (●) and SIVmac239Δnef (○). Data is expressed as SIV RNA copies/ml of plasma.

To evaluate the effects of tenofovir in macaques infected with SIVmac239Δnef, 3 of 5 macaques were given daily subcutaneous injections of tenofovir at a dose of 30 mg/kg for 4 weeks. Previous studies have demonstrated significant suppression of virulent SIV during both acute [21,22] and chronic [20,23] infection in both juvenile and adult macaques at similar dosing levels. In our hands, tenofovir rapidly reduced plasma viral load within 24 hrs of initiation of treatment in an adult macaque infected with pathogenic SIVmac251 (1484, Fig. 3A). The same dosing regimen was also found to reduce replication of SIVmac239Δnef by 2 logs_10 _(1498, Fig. 3B), indicating that tenofovir is effective at inhibiting replication of *nef*-deleted SIV. Less pronounced changes in viral load were observed in two other macaques with intrinsically low baseline levels of plasma SIVmac239Δnef RNA (1488, 1514) (Fig. 4). Two additional macaques infected with SIVmac239Δnef (1494, 1512) did not receive tenofovir and served as untreated controls. Plasma viremia in these animals ranged from < 50 to 1.7 × 10^3 ^SIV RNA copies/ml plasma at baseline with no consistent changes in viral load over the 4-week period corresponding to the interval of tenofovir treatment (Fig. 4).

### Outcome of challenge with drug-resistant SIVmac055

After 4 weeks of drug intervention, the tenofovir-treated and control macaques were intravenously challenged with 10^4 ^TCID_50 _of SIVmac055. Replication of SIVmac055 and SIVmac239Δnef were monitored by three methods: 1) allele-specific real-time PCR with molecular beacons to discriminate between the two viruses, 2) PCR to detect wild-type and *nef*-deleted alleles, and 3) PCR amplification and direct sequencing of regions of the SIV *pol *gene to identify drug resistance mutations within RT.

**Figure 5:**
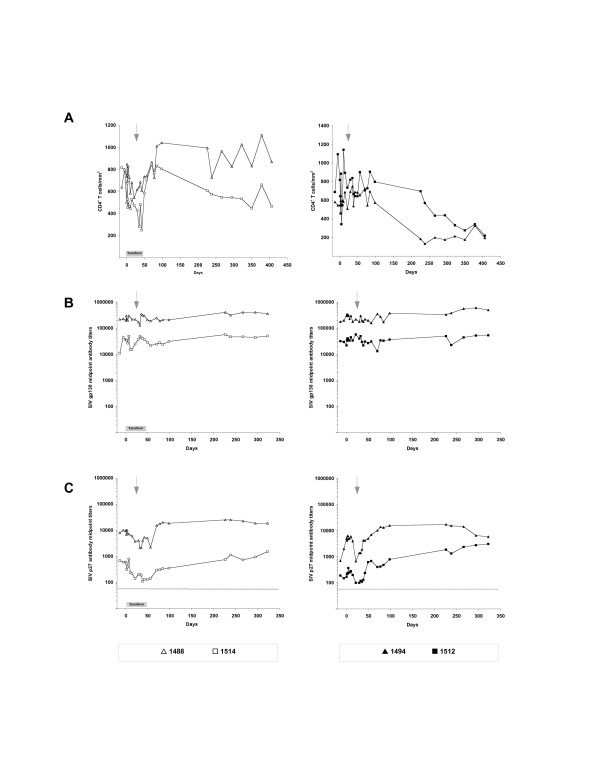
**CD4^+ ^T cell counts and SIV-specific antibody responses in tenofovir-treated and untreated macaques infected with SIVmac239Δnef**. Tenofovir-treated (1488, 1514) and untreated (1494, 1512) macaques infected with SIVmac239Δnef were challenged intravenously with SIVmac055 and monitored for (A) CD4^+ ^T cell counts, (B) SIV gp130 antibody responses, and (C) SIV p27 antibody responses for approximately one year. Tenofovir treatment was given on days 0–42. Intravenous inoculation of SIVmac055 occurred on day 28 (arrow).

Evaluation of virus replication in the two untreated control macaques (1494, 1512) revealed the presence of SIVmac055 in both animals within several weeks of intravenous challenge (Table 3). Sequence and PCR analyses of *nef *alleles demonstrated that, in the first 2 weeks after challenge, the replicating viral population in macaque 1494 was predominantly SIVmac239Δnef. However, 6 to 7 weeks after challenge, viral RNA sequences consistent with SIVmac055 were detected. By 10 weeks and thereafter, viral RNA contained wild-type *nef *alleles and SIVmac239Δnef *pol *sequences suggesting that virus recombination between SIVmac055 and SIVmac239Δnef had occurred in this animal. To confirm these results, a 7.0 kb fragment (nucleotides 2904 to 9894 [24]) spanning SIV *pol *through *nef *was cloned and sequenced. Multiple clones demonstrated SIVmac239 *pol *sequences and wild-type *nef *alleles (Table 3). Macaque 1494 was euthanized approximately 2 years after SIVmac055 challenge with clinical symptoms of severe enterocolitis and diarrhea, and marked loss of CD4^+ ^T cells (Fig. 5A).

**Table 3:** Detection of SIVmac239Δnef and SIVmac055 following challenge with SIVmac055

		**Weeks after challenge with SIVmac055**										
		
**Macaque**	**gene**	**1**	**2**	**6**	**7**	**10**	**29**	**33**	**37**	**41**	**45**	**41**^a^
1494	pol	239	239	055	055	239	239	239	239	239	239	239
	nef	Δnef	Δnef	wt	wt	wt	wt	wt	wt	wt	wt	wt
1512	pol		239			055	055	055	055	055	055	
	nef		Δnef			wt	wt	wt	wt	wt	wt	
1488	pol	239	239					239				
	nef	Δnef	Δnef					Δnef				
1514	pol	239				239		239				
	nef	Δnef				Δnef		Δnef				

^a ^Cloned 7 kb fragment

The other untreated macaque (1512) also had evidence of SIVmac055 infection with low levels of virus replication. Sequenced *pol *genes revealed drug resistance mutations in RT consistent with SIVmac055 (Table 3). An additional K64R mutation in RT was also found. Despite low levels of viral replication, CD4^+ ^T cells steadily declined after 8 months (Fig. 5A), and this macaque died approximately 18 months after SIVmac055 challenge due to a lung infarction caused by massive thrombosis.

Three additional macaques infected with SIVmac239Δnef (1488, 1498, 1514) were treated with tenofovir as described and monitored for viral replication after challenge with SIVmac055. Unexpectedly, one of the tenofovir-treated animals (1498) died within hours of the SIVmac055 challenge. Autopsy revealed severe hepatic degeneration consistent with an idiosyncratic drug reaction to tenofovir and reduced clearance of the anesthetics.

The two remaining tenofovir-treated animals (1488 and 1514) continued to receive daily drug treatment for an additional 2 weeks after SIVmac055 challenge with no adverse events. In these macaques, SIVmac055 RNA was undetectable and remained so throughout a year of follow-up. Several blips of viremia occurred, and analyses of both viral RNA and DNA demonstrated persistence of *nef *deletions and *pol *sequences consistent with SIVmac239Δnef (Fig. 4). We were unable to generate PCR amplicons containing either wild-type *nef *or SIVmac055 *pol *sequences despite multiple attempts using different peripheral blood samples from these macaques (Table 3). While CD4^+ ^T cells transiently dropped in both macaques during tenofovir treatment, cell counts recovered after treatment and remained within the normal range throughout follow-up (Fig. 5A). Both animals remained healthy for more than three years after SIVmac055 challenge, at which time the study was completed.

### SIV-specific immune responses

SIV-specific antibodies and CD8^+ ^T cell responses were evaluated in both tenofovir-treated and control macaques before and after challenge with SIVmac055. Antibodies to both SIV gp130 and p27 antigens were detected in all animals prior to SIVmac055 challenge. Titers of anti-gp130 antibodies did not change significantly in any of the macaques as a consequence of tenofovir treatment and similar patterns of responses were seen in both treated and control animals (Fig. 5B). Antibody titers to SIV gp130 varied by up to 1 log_10 _among the macaques, but these differences were not associated with tenofovir treatment, viral load, or clinical outcome following SIVmac055 challenge.

Antibody titers to SIV p27 were lower overall as compared to gp130 antibody titers, but again showed no consistent relationship to tenofovir treatment. A transient drop in anti-p27 antibodies occurred in all the macaques following SIVmac055 challenge, but these titers subsequently increased to pre-challenge levels and were not clearly associated with adverse outcome (Fig. 5C).

SIV-specific CD8^+ ^T cell responses were also assessed in the tenofovir-treated (1488, 1514) and untreated (1494, 1512) macaques (Table 4). ELISPOT assays were used to measure IFN-γ secretion using recombinant vaccinia virus (rVV)-vectors expressing SIV Gag, Pol, Env and Nef proteins [25]. Macaque peripheral blood mononuclear cells (PBMC) were assayed at baseline (day -35), two weeks after initiation of tenofovir treatment (day -15) and 9 days after challenge with SIVmac055 (day 9) (Table 4). The number of spot-forming cells (SFC) to SIV antigens initially increased in 3 of 4 macaques during the period spanning tenofovir treatment (day -35 to day -15), but these increases were observed for both tenofovir-treated (1488, 1514) and untreated (1494) macaques. SFC generally decreased following SIVmac055 challenge (day -15 to day 9) but again no consistent differences were observed between tenofovir-treated and control animals. During long-term follow-up, two protected macaques (1488, 1514) and one unprotected macaque that controlled SIVmac055 replication (1512) experienced an increase in the number of SFC approximately nine months after the challenge with SIVmac055 (day 265). The remaining untreated macaque (1494), which developed AIDS within 2 years after SIVmac055 challenge, experienced a decrease in CD8^+ ^T cell responses at nine months.

**Table 4:** SIV-specific CD8^+ ^T cell responses before and after SIVmac055 challenge

**Macaque**	**Day^a^**	**SIVenv^b^**	**SIVgag^b^**	**SIVpol^b^**	**SIVnef^b^**	**Tenofovir^c^**
1494	(-35)	60	240	155	95	No
	(-15)	160	520	220	205	
	9	45	255	115	120	
	265	35	180	40	90	
1512	(-35)	30	290	190	50	No
	(-15)	25	105	115	35	
	9	40	95	60	15	
	265	475	545	440	349	
1488	(-35)	620	170	10	50	Yes
	(-15)	2365	510	70	80	
	9	1533	458	83	168	
	265	3305	1335	205	160	
1514	(-35)	85	190	15	15	Yes
	(-15)	245	380	60	30	
	9	65	85	25	0	
	265	265	535	55	70	

^a^Days before and after SIVmac055 challenge

^b^Data is expressed as the number of spot-forming cells (SFC) per 10^6 ^PBMC

^c^Tenofovir treatment for a total of 6 weeks at a dose of 30 mg/kg of body weight

Overall, macaques infected with SIVmac239Δnef exhibited SIV-specific antibodies and CD8^+ ^T cell responses to multiple viral antigens. However, these responses did not differ significantly between tenofovir-treated and untreated animals. While we were unable to assay for functional neutralizing antibodies and cytotoxic T lymphocyte (CTL) responses in this study due to sample limitations, we have previously shown that macaques infected with SIVmac239Δnef develop both SIV-specific neutralizing antibodies [16] and functional CD8^+ ^T cell responses [17], and these responses may persist over time.

## Discussion

The results of this study provide further support for the concept that antiretroviral drug treatment augmented by virus-specific immunity can prevent replication of drug-resistant virus during chronic SIV infection. The animals used in this study were previously found to have robust and broadly reactive SIV-specific immune responses induced by infection with live, attenuated SIV [16]. These animals remained clinically healthy and intermittently viremic for several years with low-level replication of SIVmac239Δnef, thus providing an opportunity to evaluate the impact of drug treatment and suppression of drug-resistant virus in macaques with chronic SIV infection.

Tenofovir has been shown to mediate potent and durable suppression of virulent SIV in both adult and neonatal macaques [20,21,23,26-32]. When administered early during the acute phase of SIV infection, macaques treated with tenofovir have significantly reduced viremia and improved clinical survival as compared to untreated animals [12]. The impact of tenofovir in suppressing viral replication is due in part to the synergistic action of CD8^+ ^T cells, which control replication of SIV during both acute and chronic phases of infection [17,33,34], suggesting that antiviral immunity plays a key role in determining the success of antiretroviral drugs [14]. Interestingly, when CD8^+ ^T cells are depleted *in vivo *in macaques on long-term tenofovir therapy, viral rebound is associated with the presence of SIV variants harboring drug resistance mutations and reduced sensitivity to tenofovir [14], suggesting that antiviral immunity can suppress replication of drug-resistant virus.

Our results are consistent with this observation in macaques with strong anti-viral immune responses induced by chronic infection with live, attenuated SIV. Previously we demonstrated that *in vivo *depletion of CD8^+ ^T cells in macaques infected with SIVmac239Δnef results in an increase in viremia, which is temporally controlled with restoration of the CD8^+ ^T cell population, thus supporting the role of these cells in suppressing endogenous virus replication [17]. Furthermore, we have shown that this transient increase in endogenous SIV antigenaemia can enhance virus-specific immunity and is associated with protection from virulent SIVmac055 challenge [35]. In the present study, we found that SIV-specific immune responses in macaques chronically infected with SIVmac239Δnef were alone unable to prevent replication of drug-resistant SIVmac055 in untreated macaques. This may be due in part to waning of SIV-specific immune responses over time coupled with ongoing replication of live, attenuated SIV with increased pathogenicity [36].

But when combined with a short-course of tenofovir, replication of SIVmac055 was inhibited, suggesting that antiviral immunity can be effective when acting in concert with ART to suppress replication of drug-resistant virus. The macaques in this study were treated with tenofovir for 4 weeks prior to challenge with drug-resistant SIV. While tenofovir has direct antiviral effects against SIV through potent inhibition of the viral RT, it is also known to stimulate secretion of a number of immunomodulatory cytokines and chemokines, including interleukin-1β (IL-1β), IL-10, tumor necrosis factor-α, RANTES and macrophage inflammatory protein-1α [37,38]. These factors have both inhibitory and stimulatory effects on HIV-1 replication [39], in addition to their role in regulating immune cell function. Treatment with tenofovir may, therefore, augment antiviral immunity by stimulating immunomodulatory factors and creating an environment less permissive for replication of drug-resistant strains, particularly those with reduced fitness compared to wild-type [5].

Our current findings suggest that replication of drug-resistant SIVmac055, which harbors several resistance and compensatory mutations in RT, can be inhibited in immunocompetent animals receiving concurrent therapeutic intervention with tenofovir. SIVmac055 is an uncloned virus stock derived from an infant macaque infected with SIVmac251 and receiving long-term therapy with tenofovir and is therefore genotypically closely related to SIVmac251 [40]. This raises the question as to why the untreated macaques failed to prevent infection with SIVmac055, when they were previously protected from challenge with SIVmac251 [16]. One possible explanation is the presence of variants in the SIVmac055 stock that escape immune recognition due to mutations in CD8^+ ^T cell epitopes. However, in untreated macaques that failed to be protected, replicating virus contained resistance mutations consistent with SIVmac055 indicating persistence of the drug-resistant genotype. It is known that cytotoxic T lymphocytes (CTL) from HIV-1 infected patients on antiretroviral therapy continue to respond *in vitro *to peptides containing drug resistance mutations [9,41], and this may have contributed to control of SIVmac055 replication in the tenofovir-treated macaques, but may have been insufficient to block SIVmac055 replication in the untreated animals.

Tenofovir was withdrawn 2 weeks after SIVmac055 challenge in the treated macaques with no evidence of viral rebound, and only intermittent detection of SIVmac239Δnef, suggesting immune-mediated control of viremia is sustained in the absence of further drug intervention. No evidence was found for SIVmac055 infection in the tenofovir-treated macaques, in contrast to naïve control animals that received pre-exposure prophylaxis with tenofovir and experienced significant rebound of SIVmac055 viremia and clinical progression after drug was withdrawn. Taken together, these data indicate that SIVmac055 is able to infect and replicate in the presence of tenofovir during primary infection, and immune control of viremia is not sustained when drug is withdrawn. Similarly, macaques infected with SIVmac239Δnef that developed SIV-specific immune responses, but were not treated with tenofovir, were unable to prevent infection and replication of SIVmac055 indicating that antiviral immunity alone is insufficient to suppress drug-resistant SIV. Only those macaques that had both demonstrable antiviral immunity to SIV and short-term tenofovir treatment were able to prevent SIVmac055 infection, and these animals sustained low levels of viremia with SIVmac239Δnef for over three years after the drug was withdrawn.

## Conclusion

In humans, potent suppression of chronic HIV-1 replication is achieved through therapeutic administration of antiretroviral drugs, which can reduce viremia often to undetectable levels for sustained periods, and can lead to partial restoration of CD4^+ ^T cells and immune function [1-3]. The extent to which antiviral immune responses suppress the emergence of drug-resistant HIV-1 in humans *in vivo *is unknown, but our data in non-human primates support the idea that immune mechanisms contribute significantly to suppression of drug-resistant virus during chronic infection, and can augment the efficacy of ART. Strategies that seek to combine antiretroviral therapy with stimulation of HIV-1 specific immune responses [11], particularly CD8^+ ^T cell responses, may be effective at treating HIV-1 infection and preventing therapeutic failure associated with the emergence of drug-resistant virus.

## Methods

### Rhesus macaques

A total of nine adult rhesus macaques (*Macaca mulatta*) were used in this study. Five were infected with SIVmac239Δnef (kindly provided by Dr. Ronald Desrosiers, New England Primate Research Center, Harvard Medical School, Southborough, MA) by intravenous inoculation of 4 × 10^3 ^50% tissue-culture infective doses (TCID_50_) as part of a larger vaccine study [16]. The animals were monitored for SIV-specific humoral and cellular immune responses, and for the ability to resist challenge with pathogenic SIVmac251 over a follow-up period of three years [16,42]. No evidence was found for infection with SIVmac251 in any of the animals based on repeated PCR evaluation of both peripheral blood mononuclear cells (PBMC) and lymph nodes [16], including analyses done immediately prior to initiation of this study. Conversely, SIVmac239Δnef was intermittently detected at low levels in all five macaques throughout the 3-year follow-up period. SIV-specific humoral and cellular immune responses were measured for each macaque to evaluate the correlates of immune protection associated with resistance to SIVmac251 challenge, and each of the macaques was found to have broad SIV-specific antibodies and CD8^+ ^T cell responses [16].

Four naïve adult rhesus macaques were additionally used as controls to evaluate infection and replication of drug-resistant SIVmac055. The control macaques were negative for type D retrovirus, and for SIV RNA and DNA prior to initiation of this study. All animal protocols were approved by the International Animal Care and Use Committee at the Tulane Regional Primate Research Center.

### Tenofovir treatment

Control and SIV-infected rhesus macaques were treated with tenofovir (PMPA, kindly provided by Dr. Norbert Bischofsberger, Gilead Science, Foster City, CA) at a dose of 30 mg/kg per body weight for six weeks by daily subcutaneous injection.

### Drug-resistant SIVmac055

SIVmac055 (kindly provided by Dr. Koen van Rompay, California Regional Primate Research Center, University of California, Davis, CA) was first isolated from an infant macaque infected with SIVmac251 and receiving prolonged therapy with tenofovir. SIVmac055 exhibits a 5-fold increased resistance to tenofovir *in vitro *associated with a K65R mutation in the viral reverse transcriptase [20,40]. A stock of SIVmac055 was prepared in rhesus PBMC and titered on CEM×174 cells. Macaques were intravenously inoculated with 10^4 ^TCID_50 _of SIVmac055 and virus replication was monitored by real-time PCR and molecular beacons designed to differentially quantify SIVmac239Δnef and SIVmac055 RNA in plasma and PBMC as previously described [17].

To further investigate the genomic characteristics of replicating viruses, SIV *pol *and *nef *genes were analyzed by cloning and sequencing as described before [35].

### ELISPOT assay, ELISA, and flow cytometry

The ELISPOT assay used for detection of IFN-γ secretion by CD8^+ ^T cells was modified from Larsson et al. [25] and previously described [35,43]. Antibodies to SIV gp130 and p27 in macaque plasma samples were detected by standard ELISA methods as described [16,35]. CD4 and CD8 T-cell subsets from whole blood collected in EDTA were analyzed by using anti-CD3 (Biosource International, Camarillo, CA), anti-CD4 and anti-CD8 (anti-Leu3a and anti-Leu2a, respectively; Becton Dickinson, San Jose, CA) as previously described [16].

## Competing interests

The author(s) declare that they have no competing interests.

## Authors' contributions

KJM carried out SIV viral load quantification and sequence analyses, participated in the conception and design of the study, and drafted the manuscript. JMB carried out immunoassays for quantifying SIV antibody responses. AG coordinated all live animal work including tenofovir treatment and sample collection. PM participated in the conceptual design of the study. DFN conducted CD8^+ ^T cell assays and participated in the design of the study. RIC participated in the design of the study and helped to draft the manuscript. All authors read and approved the final manuscript.
